# Classification of Adverse Events Following Surgery in Patients With Diffuse Lower-Grade Gliomas

**DOI:** 10.3389/fonc.2021.792878

**Published:** 2021-12-21

**Authors:** Tomás Gómez Vecchio, Alba Corell, Dongni Buvarp, Isabelle Rydén, Anja Smits, Asgeir S. Jakola

**Affiliations:** ^1^ Department of Clinical Neuroscience, Institute of Neuroscience and Physiology, Sahlgrenska Academy, University of Gothenburg, Gothenburg, Sweden; ^2^ Department of Neurosurgery, Sahlgrenska University Hospital, Gothenburg, Sweden; ^3^ Department of Neuroscience, Uppsala University, Uppsala, Sweden

**Keywords:** glioma grade 2, glioma grade 3, neurosurgery, postoperative complications, classification, health-related quality of life, patient-centered care

## Abstract

**Background:**

Recently, the Therapy-Disability-Neurology (TDN) was introduced as a multidimensional reporting system to detect adverse events in neurosurgery. The aim of this study was to compare the novel TDN score with the Landriel*–*Ibanez classification (LIC) grade in a large cohort of patients with diffuse lower-grade glioma (dLGG). Since the TDN score lacks validation against patient-reported outcomes, we described health-related quality of life (HRQoL) change in relation to TDN scores in a subset of patients.

**Methods:**

We screened adult patients with a surgically treated dLGG World Health Organization (WHO) grade 2 and 3 between 2010 and 2020. Up until 2017, it consists of a retrospective cohort (*n* = 158). From 2017 and onwards, HRQoL was registered using EuroQoL-5-dimension, three levels of response (EQ-5D 3L) questionnaire at baseline and 3 months follow-up, in a prospectively recruited cohort (*n* = 102). Both the LIC grade and TDN score were used to classify adverse events.

**Results:**

In total, 231 patients were included. In 110/231 (47.6%) of the surgical procedures, a postoperative complication was registered. When comparing the TDN score to LIC grades, only a minor shift towards complications of higher order could be observed. EQ-5D 3L was reported for 45 patients. Patients with complications related to surgery had pre- to postoperative changes in EQ-5D 3L index values (*n* = 27; mean 0.03, 95% CI −0.06 to 0.11) that were comparable to patients without complications (*n* = 18; mean −0.06, 95% CI −0.21 to 0.08). In contrast, patients with new-onset neurological deficit had a deterioration in HRQoL at follow-up, with a mean change in the EQ-5D 3L index value of 0.11 (*n* = 13, 95% CI 0.0 to 0.22) compared to −0.06 (*n* = 32, 95% CI −0.15 to 0.03) for all other patients.

**Conclusions:**

In patients with dLGG, TDN scores compared to the standard LIC tend to capture more adverse events of higher order. There was no clear relation between TDN severity and HRQoL. However, new-onset neurological deficit caused impairment in HRQoL. For the TDN score to better align with patient-reported outcomes, more emphasis on neurological deficit and function should be considered.

## Introduction

A standardized reporting system for adverse events has been much wanted in neurosurgery. The Clavien–Dindo (1) and its adaptation the Landriel–Ibanez classification (LIC) (2) systems have been more commonly used in recent literature. Both scales classify adverse events relying on the therapy used to treat the complication. Such classifications were criticized since the kind of treatment required by a specific complication may not always correlate with the patient’s health status at discharge and follow-up (3). This is especially true for new neurological deficits following neurosurgery that are typically left untreated, hence being classified as a mild complication. Recently, the Therapy-Disability-Neurology (TDN) score was proposed and suggested to better capture the neurological aspects of complications (4). In TDN, adverse events are graded in relation to the therapy, disability, and neurological deficits that are involved. This system uses Clavien–Dindo and LIC as fundaments, but also adds function with the modified Rankin Scale (mRS) and neurological deficit to the classification.

The novel TDN score was initially validated against Karnofsky performance status scale (KPS). This can be criticized since mRS and KPS have similar prognostic value (5), besides being both a clinical reported outcome. A better calibration, or at least a valid supplementation, may be to add patient-reported outcome measures (PROMs) to the TDN. Multidimensional PROMs such as health-related quality of life (HRQoL) are useful to determine patients’ needs in a broader setting with a patient-centered approach. It has been shown that in addition to the mRS, PROMs may play an important role in the assessment of health status in clinical practice (6, 7).

Still, TDN is a promising multidimensional and patient-centered approach to the classification of the severity of adverse events in neurosurgery. A standardized reporting system would allow for monitoring and comparison, where the goal of benchmarking and transparency would ultimately improve the quality of care for patients undergoing surgery. However, more studies are needed to evaluate the differences between the traditional reporting and the TDN score. Of particular interest in this respect are the questions how often the inclusion of mRS and neurological deficits significantly changes the classification and how the complication grades relate to PROMs.

In this study, we aimed to compare the novel TDN score with the LIC grade in a large cohort of patients with diffuse lower-grade glioma (dLGG), to establish the relationship between these two measures. In a subset of patients, we explored the relation between the abovementioned scales (LIC grade and TDN score) and HRQoL following neurosurgical management.

## Materials and Methods

### Recruitment

All patients were recruited at our neurosurgical department, which covers a population of approximately 1.7 million inhabitants in a system with referrals based on area of residence. The department manages all patients requiring a neurosurgical procedure due to an intracranial lesion in the region of Västra Götaland, Sweden.

**Figure 1:**
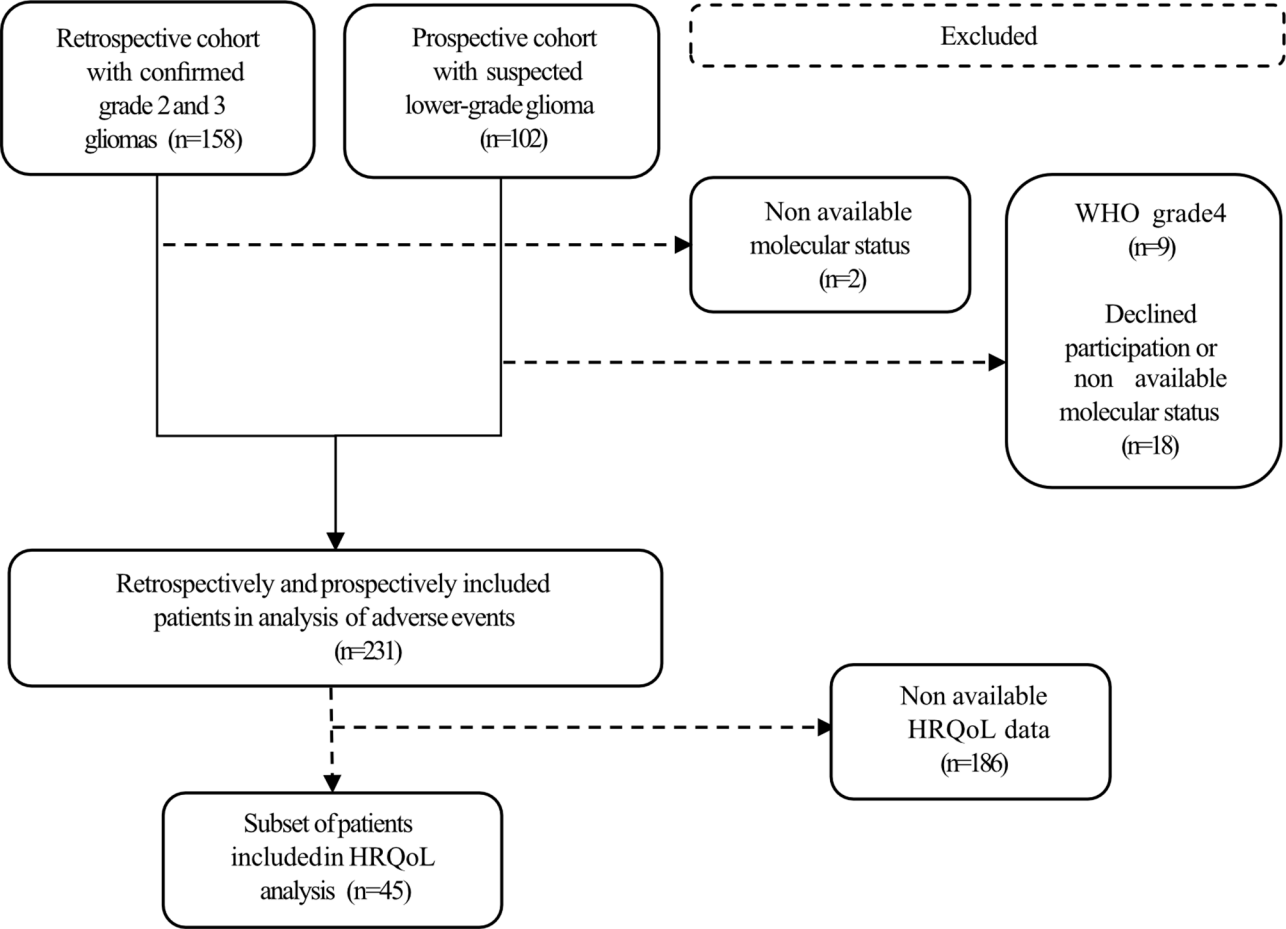
Flow chart of included cases.

Data were derived from two cohort studies, one retrospective and one prospectively recruited (**Figure 1**). The *retrospective* cohort was obtained using the electronical health records (EHR), pathology database, and operation logs. Patients were ≥18 years old with histopathological verified supratentorial dLGG classified according to the 2007 World Health Organization (WHO) classification of tumors of the central nervous system (8) and graded as grade 2 or 3. Patients who underwent biopsy or tumor resection during the period January 2010 through December 2016 were identified.

The *prospectively recruited* cohort consists of patients ≥18 years old with radiologically suspected dLGG based on MRI scan(s). These patients underwent biopsy or tumor resection during the period January 2017 through December 2020. As part of pre-operative work-up and approximately 3 months postoperatively, these patients were invited to provide quality-of-life measurement in terms of EQ-5D 3L. Subsequently, only patients with dLGG classified according to the WHO 2016 classification (9) as grade 2 or grade 3 were selected for the study.

### Assessment of Molecular Status

Immunohistochemistry staining for *isocitrate dehydrogenase* 1 R132H (*IDH*) mutant protein was performed as the initial step in assessment of *IDH* mutation. Negative immunohistochemistry analyses were subsequently tested with next-generation sequencing to detect rarer *IDH* mutations (10). Codeletion of chromosomal arms 1p and 19q was evaluated with fluorescence *in situ* hybridization, multiplex ligation-dependent probe amplification or evaluation from methylation arrays as reported previously (11). A minority of the retrospective material was not evaluated according to WHO 2016 due to lack of tissue and therefore excluded from the study. The 2021 fifth edition of the WHO Classification of the Tumors of the Central Nervous System (WHO 2021) was not available during the design of the study. As a result, all remaining material, including *IDH* wild-type dLGG where the majority show clinical features of glioblastoma and are currently assessed as glioblastoma according to WHO 2021, was classified according to WHO 2016 for this study (12).

### Measures

KPS is an ordinal scale designed to measure levels of patient activity and medical requirements. Patients are classified into 11 categories from 100 (no evidence of disease) to 0 (dead) (5, 13). KPS at admission was retrospectively scored based on data extracted from EHR. The mRS was originally designed for stroke patients; it focuses on patient disabilities, and patients are classified on 7 categories from 0 (no symptoms) to 6 (dead) (5, 14). For all patients, the mRS was retrospectively estimated from the EHR at follow-up visit (1–3 months postoperative) by clinicians (AJ and DB). To assess whether mRS was affected by surgical complications in patients registering adverse events, mRS at follow-up was qualitatively and retrospectively estimated from EHR.

Adverse events related to post-operative complications were evaluated using the LIC. LIC focuses on general postoperative morbidity using a four-grade severity scale based on the therapy administered to treat a postoperative adverse event within 30 days of surgery; it also considers whether the complication is medical or surgical (2). Complications were recorded based on EHR.

Neurological deficit and any information concerning seizures and seizure control was routinely assessed at admission, discharge, and follow-up at the neurosurgical department. Also based on EHR, neurological deficits recorded included motor, language, cognitive, and visual domains. Any post-operative new or worsened neurological deficit, including transient or suspected ones like the supplementary motor area syndrome, was registered. A deficit was considered permanent if deterioration compared to baseline was still present at 3 months, even if significant recovery had occurred.

Anatomical magnetic resonance imaging (MRI) T2-weighted image (T2) or fluid-attenuated inversion-recovery (FLAIR) sequences were used to assess tumor volume using software 3D Slicer (15) according to our previously reported method (16). Multifocal lesions were classified according to the largest tumor. Main tumor location and presumed eloquent brain areas were routinely identified as part of the preoperative work, and both were recorded based on EHR. Location taxonomy followed the anatomical lobe mainly involved by the lesion. Presumed eloquent brain areas were identified following the areas listed in the University of California San Francisco classification system (17).

**Figure 2:**
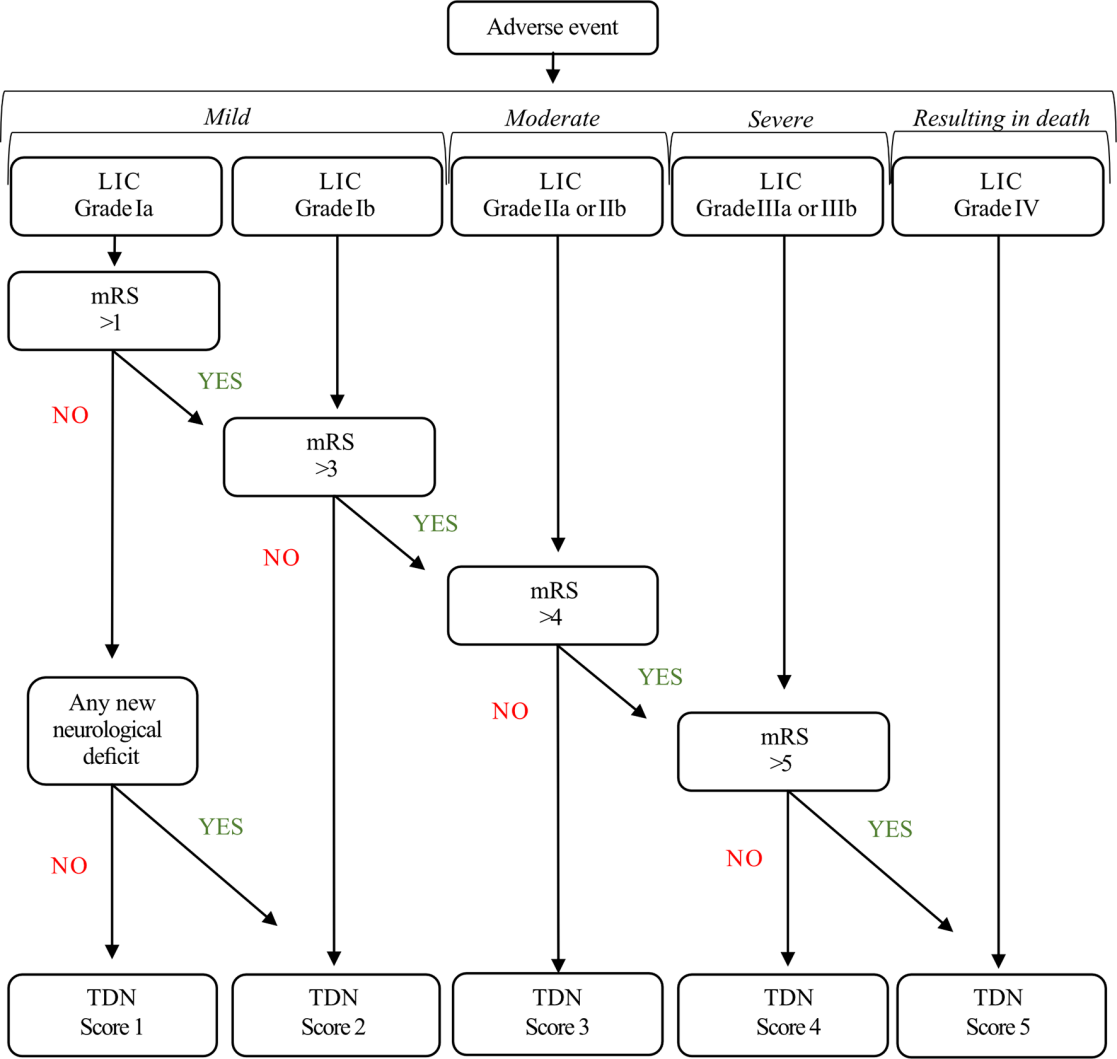
TDN algorithm.

TDN grades were calculated following the TDN criteria (4), here referred to as TDN scores. Adverse events were ordered in relation to the therapy, disability, and neurological deficits they involved. Therapy was evaluated using the LIC; Disability was assessed with the mRS at follow-up (mRS was not considered for TDN classification if it was affected by documented tumor progression); Neurological deficit was assessed using a binary definition for any new or worsened neurological deficit following surgery. According to TDN criteria, the dimensions of Disability and Neurology were only considered for TDN scoring when their deterioration resulted from the adverse event (**Figure 2**).

HRQoL was measured with EuroQoL-5-dimension, three levels of response (EQ-5D 3L) questionnaire (18). Patients completed the EQ-5D 3L at the time of first visit to the outpatient clinic and at follow-up approximately 3 months postoperatively. EQ5D 3L was only available in parts of the prospective cohort. The results of EQ-5D 3L questionnaire were transferred to a utility index ranging from −0.594 to 1 (19), where higher scores indicated better quality of life. A minimal clinical important difference (MCID) of ±0.14, previously reported in patients undergoing glioma surgery, was used for this study (20). Change in EQ-5D3L index value was calculated subtracting the follow-up value from the preoperative value. Negative values (postoperative better than preoperative) indicated improvement; positive values indicated a decline.

### Statistical Analysis

All analyses were carried out in IBM SPSS version 28 (IBM Corp., Armonk, NY, US). Central tendencies for descriptive statistics are presented with either percentages, means with standard deviation (SD) or 95% confidence intervals (CI), or medians with first and third quartile (Q1, Q3). Statistical significance level was set to *p* < 0.05. All tests were 2 sided. Comparisons between groups were conducted with unpaired *t*-test, Mann–Whitney *U* test, Pearson *χ* ^2^, or Fisher’s exact test as appropriate. Collinearity was assessed with Pearson correlation, ANOVA, and Cramer test between covariables, covariables and factors, and between factors, respectively. Collinearity between variables was set to values higher than 0.80 for Pearson’s eta, 0.64 for ANOVA’s eta squared, and 0.80 for Cramer’s V. Multivariable logistic regression was used with complications related to surgery as response. Age, sex, preoperative KPS, epilepsy, neurological deficits at admission (motor, cognitive, visual, and language), type of neurological intervention, tumor classification, main tumor location, tumor volume, and preoperative eloquence were used as dependent variables. Additional Sankey diagram, bar plots, and box plots were generated using Python programming language version 3.8.3 (Python Software Foundation, Delaware, US).

## Results

### Patient Characteristics

A total of 260 patients were screened for inclusion. Two patients in the retrospective cohort were excluded due to lack of tissue. From the prospectively recruited cohort, 27 patients were excluded because final histopathological diagnosis was other than dLGG grade 2 or grade 3, or because patients declined to participate in the study (**Figure 1**).

A total of 231 patients were included in the study. The mean age at surgery was 48.3 years (SD 14.5), and 134 patients (58.0%) were males. There were 69 patients (29.9%) with oligodendrogliomas, 75 (32.5%) with astrocytoma *IDH*-mutant, and 87 (37.6%) with astrocytoma *IDH* wild type. The distribution of grade showed that 119 patients (51.5%) had tumors of WHO grade 2 and 112 patients (48.5%) of WHO grade 3. Within 3 months of surgery, a total of 46/231 patients (19.9%) started chemotherapy only (either temozolomide or procarbazine-lomustine-vincristine), and 52/231 patients (22.5%) had started radiotherapy only, and 61/231 patients (26.4%) had started both chemotherapy and radiotherapy.

At admission, 157 patients (68%) reported a history of seizures. From surgery to the 3 months follow-up, five of these patients (2.2% of total cohort) had worsening of seizures. During the same period, three patients (1.3%) had first-onset seizures. All worsened and first-onset seizures occurring withing 30 days of surgery were scored according to LIC grading. The proportion of any neurological deterioration post-operatively was 89/231 (38.5%). Deficit in more than one function occurred in 36/231 patients (15.6%). Also, out of 89 patients with deficits, 40 patients (44.9%) had complete recovery at 3 months postoperatively. A detailed list of patient characteristics and clinical variables is provided in **Table 1**.

**Table 1:** Patient characteristics and clinical variables (*N* = 231).

Variable	Study sample
Age at surgery, mean (SD)	48.3 (14.5)
Female, *n* (%)	97 (42.0)
KPS^1^ at admission, median (Q1, Q3)	90 (80, 90)
WHO 2016 classification, *n* (%)	
Oligodendroglioma, WHO grade 2	36 (15.6)
Oligodendroglioma, WHO grade 3	33 (14.3)
Diffuse astrocytoma, IDH-mutant, WHO grade 2	36 (15.6)
Astrocytoma, IDH-mutant, WHO grade 3	39 (16.9)
Diffuse astrocytic glioma, IDH-wildtype, WHO grade 2	47 (20.3)
Diffuse astrocytic glioma, IDH-wildtype, WHO grade 3	40 (17.3)
Seizure, *n* (%)	157 (68.0)
Neurological deficit at admission, *n* (%)	
Motor	28 (12.1)
Cognitive	39 (16.9)
Visual	12 (5.2)
Language	28 (12.1)
Any neurological deficit	76 (32.9)
Type of neurosurgical intervention, *n* (%)	
Tumor resection	184 (79.7)
Seizure^2^, *n* (%)	8 (3.5)
New neurological deficit ^3^, *n* (%)	
Motor	49 (21.2)
Cognitive	22 (9.5)
Visual	17 (7.4)
Language	45 (19.5)
Any new neurological deficit	89 (38.5)
* Transient deficit*	*40 (17.3)*
* Permanent deficit*	*49 (21.2)*
Deficits in more than one domain	36 (15.6)

^1^Karnofsky Performance Status Scale.

^2^New or worsened. Neither prophylactic or therapeutic use of anti-epileptic drugs were recorded for the study.

^3^New neurological deficits were defined as new or worsened from surgery to the 3-month follow-up.

### Patient Characteristics and Adverse Events Related to Surgery

**Figure 3:**
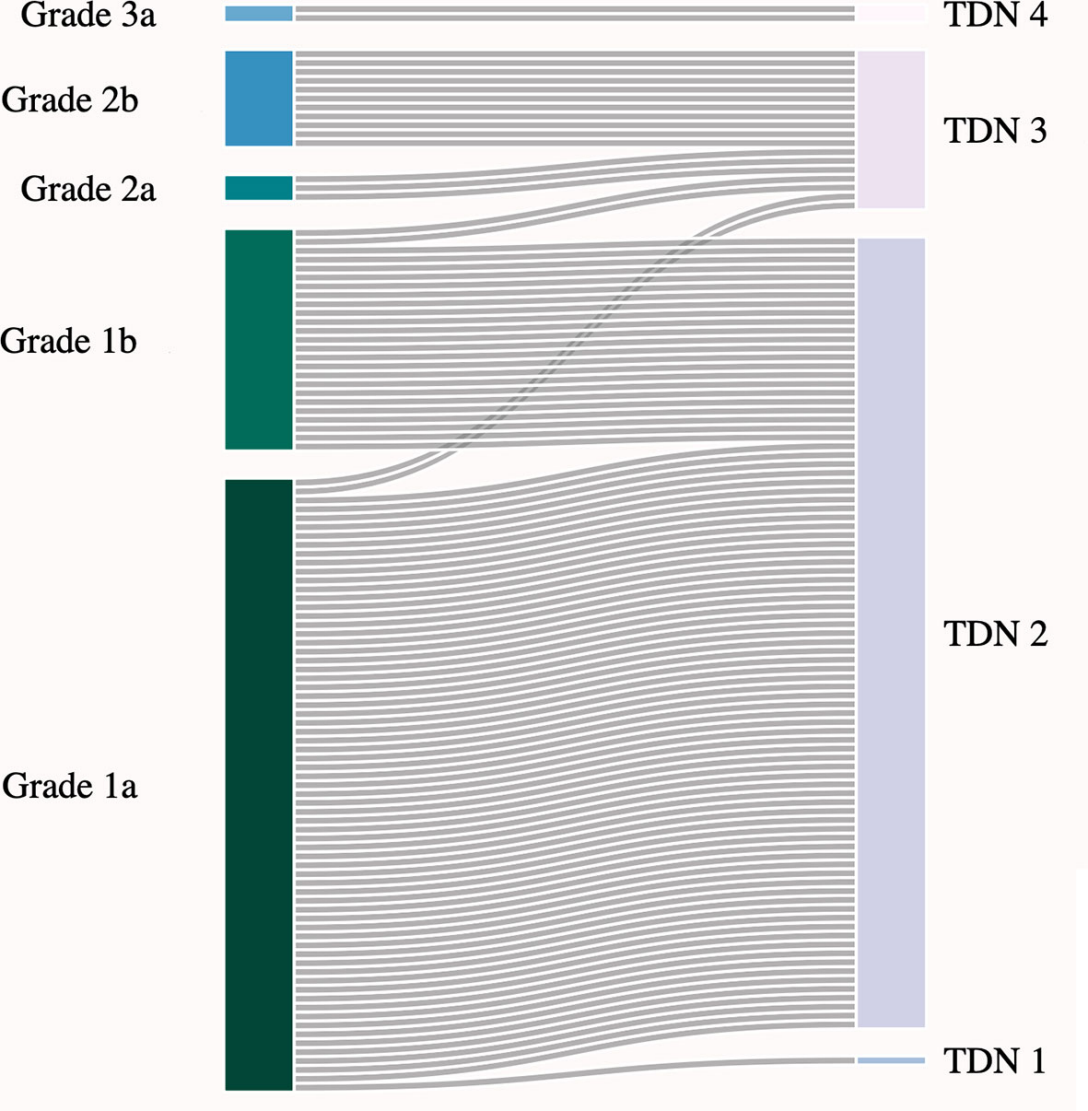
Comparison between LIC grade and TDN score (*N* = 110).

Regarding surgical outcomes, 121/231 patients (52.4%) did not present with any postoperative complications and consequently received a TDN score of 0. The remaining 110 patients with complications related to surgery were scored according to TDN criteria. In 10 out of 110 patients (9.1%) with complications, mRS measurements could not be considered for TDN scoring. Five of these ten patients had notable deterioration in mRS at follow-up due to tumor progression, which is therefore unrelated to complications following glioma surgery. For the remaining five patients, mRS was missing due to loss of follow-up. A comparison between TDN score and LIC grade results is provided in **Table 2** and **Figure 3**.

**Table 2:** Comparison between LIC grade and TDN score (*N* = 231).

Variable	Cohort (*n* = 231)	Variable	Cohort (*n* = 231)
LIC^1^, *No* (%)		TDN^2^, *No* (%)	
No complications,	121 (52.4)	No complications,	121 (52.4)
Grade Ia	69 (29.9)	Score 1	1 (0.4)
* Ia surgical//medical*	*66//3*		
Grade Ib	25 (10.8)	Score 2	89 (38.5)
* Ib surgical//medical*	*15//10*		
Grade IIa	3 (1.3)		
* IIa surgical//medical*	*2//1*	Score 3	18 (7.8)
Grade IIb	11 (4.8)		
* IIb surgical//medical*	*10//1*		
Grade IIIa	2 (0.9)	Score 4	2 (0.9)
* III surgical//medical*	*1//1*		
Grade IIIb	-		
Grade IV	-	Score 5	-
Type of complication, *No* (%)			
Medical	16 (6.9)		
Surgical	94 (40.7)		

^1^ Landriel–Ibanez classification.

^2^ Therapy-Disability-Neurology.

Patient characteristics and clinical variables in cases with and without complications related to surgery are shown in **Table 3**. Of the included variables, only preoperative cognitive deficit and type of surgery were found to be significantly differently distributed among groups of patients with and without complications. Patients with complications more often had cognitive impairment prior to surgery than patients without complications (23.6% versus 10.7%, *p* = 0.01) and more often underwent tumor resection than tumor biopsy as primary surgery strategy (90% versus 70.2%, *p* < 0.001 see **Table 3**).

**Table 3:** Patient characteristics and clinical variables in patients with and patients without complications related to surgery (*N* = 231).

Variable	No Complications (*n* = 121)	TDN^1^ (1 to 5) (*n* = 110)	*p*-value^2^
Age at surgery, mean (SD)	48.5 (14.5)	48.2 (14.6)	0.87
Female, *n* (%)	52 (43.0)	45 (40.9)	0.79
KPS^3^ at admission, median (Q1, Q3)	90 (70, 90)	90 (80, 90)	0.99
WHO 2016 classification, *n* (%)			
Oligodendroglioma, WHO grade 2	18 (14.9)	18 (16.4)	0.86
Oligodendroglioma, WHO grade 3	17 (14.0)	16 (14.5)	1.00
Diffuse astrocytoma, IDH-mutant, WHO grade 2	17 (14.0)	19 (17.3)	0.59
Astrocytoma, IDH-mutant, WHO grade 3	19 (15.7)	20 (18.2)	0.73
Diffuse astrocytic glioma, IDH-wildtype, WHO grade 2	30 (24.8)	17 (15.5)	0.10
Diffuse astrocytic glioma, IDH-wildtype, WHO grade 3	20 (16.5)	20 (18.2)	0.86
Epilepsy, *n* (%)	80 (66.1)	77 (70.0)	0.57
Neurological deficit at admission, *n* (%)			
Motor	19 (15.7)	9 (8.2)	0.11
Cognitive	13 (10.7)	26 (23.6)	0.01
Visual	8 (6.6)	4 (3.6)	0.38
Language	11 (9.1)	17 (15.5)	0.16
Any neurological deficit excluding seizures	38 (31.4)	38 (34.5)	0.68
Type of neurosurgical intervention, *n* (%)			
Tumor resection	85 (70.2)	99 (90.0)	<0.001
Main tumor location, *n* (%)			
Frontal	61 (50.4)	58 (52.7)	0.79
Temporal	34 (28.1)	32 (29.1)	0.89
Parietal	11 (9.1)	10 (9.1)	1.00
Occipital	1 (0.8)	1 (0.9)	1.00
Insular	11 (9.1)	7 (6.4)	0.47
Basal ganglia	3 (2.5)	2 (1.8)	1.00
Tumor located in eloquent regions (UCSF^4^)	79 (65.3)	78 (71.6)	0.32
Tumor volume^5^, median (Q1, Q3)	55.1 (27.6, 133.5)	54.8 (28.1, 97.8)	0.60
*Change in EQ-5L 3D index value, n (%)*	*n = 18*	*n = 27*	
* MCID^6^ change in EQ-5D 3L index value - IMPROVED*	*5 (27.8)*	*4 (14.8)*	*0.45*
* MCID change in EQ-5D 3L index value - UNCHANGED*	*10 (55.6)*	*18 (66.7)*	*0.54*
* MCID change in EQ-5D 3L index value - WORSENED*	*3 (16.7)*	*5 (18.5)*	*1.00*

^1^ Therapy-Disability-Neurology.

^2^ Statistical significance level was set to p < 0.05. All tests are 2 sided. Comparisons between groups were conducted with unpaired t-test, Mann–Whitney U-test or Fisher’s exact test as appropriate.

^3^ Karnofsky Performance Status Scale.

^4^ University of California San Francisco classification system.

^5^ Volume in cubic millimeters. One missing case due to unavailable MRI.

^6^ Minimum clinical important difference.

A multivariable logistic regression was performed to ascertain the effects of age, sex, preoperative KPS, epilepsy, neurological deficits at admission, type of neurological intervention, tumor classification, main tumor location, tumor volume, and preoperative eloquence on the likelihood that complications arise following glioma surgery. Of these, cognitive impairment at admission, surgical resection, and tumors located in eloquent regions were associated with an increased likelihood of complications following glioma surgery (respectively *p* = 0.01, *p* ≤ 0.001, and *p* = 0.01, see **Supplementary Table 1**).

### Neurological Deficit in Patients Experiencing Adverse Events Related to Surgery

In 110 patients experiencing adverse events, a total of 133 new or worsened neurological deficits were found. Of the 110 patients experiencing adverse events, 21 patients (19.1%) did not present with new or worsened neurological deficit. On average, patients scoring TDN 2 experienced more neurological deficits (112 deficits in 89 patients) and more often postoperative neurological deficit only (51/89 patients, 57%) than patients with TDN higher than 2 (21 deficits in 20 patients; and 7/20 patients, 35% respectively). A detailed list of neurological deficits in groups of patients by TDN score is shown in **Table 4**.

**Table 4:** Neurological deficits at admission and follow-up in patients by TDN score (*N* = 231).

Variable	TDN^1^ 0 *n* = 121	TDN 1 *n* = 1	TDN 2 *n* = 89	TDN 3 *n* = 18	TDN 4 *n* = 2	TDN 5 *n* = 0
New neurological deficit^2^, *n* (%)						
Motor	–	–	41 (46)	7 (39)	1 (50)	–
Cognitive	–	–	19 (21)	2 (11)	1 (50)	–
Visual	–	–	14 (16)	3 (17)	–	–
Language	–	–	38 (43)	6 (33)	1 (50)	–
*Patients with any new or worsened ND^3^*	*-*	*-*	*77 (87)*	*10 (56)*	*2 (100)*	*-*
*Patients with postoperative ND only*	*-*	*-*	*51 (57)*	*6 (33)*	*1 (50)*	*-*
*Patients with permanent ND*	*-*	*-*	*40 (45)*	*7 (39)*	*2 (100)*	*-*

^1^Therapy-Disability-Neurology.

^2^ New neurological deficits were defined as new or worsened (transient/permanent) from surgery to the 3-month follow-up.

^3^ Neurological deficits.

### Postoperative Health-Related Quality of Life

**Figure 4:**
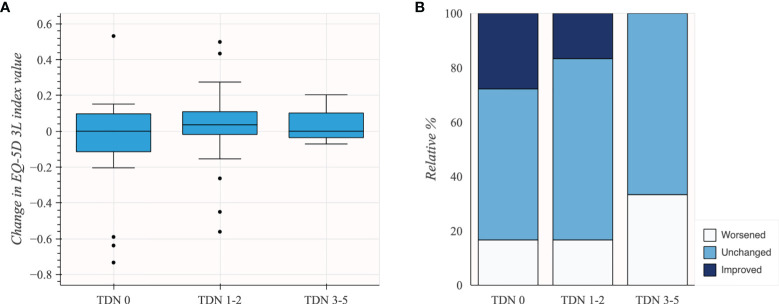
Change in the EQ-5D 3L index value in subgroups of patients based on TDN scores (*N* = 45). **(A)** Box plot illustrating distribution of change in EQ-5D 3L index values. **(B)** Stacked bar plot illustrating change in EQ-5D 3L based on MCID.

EQ-5D 3L was reported for 45 patients. The patients experiencing complications related to surgery had similar pre- to postoperative change in EQ-5D 3L index values (*n* = 27; mean 0.03; 95% CI −0.06 to 0.11) compared to patients without complications (*n* = 18; mean −0.06; 95% CI −0.21 to 0.08). Although subgroups were small, there was no apparent difference in change in EQ-5D 3L index values for TDN scores 3–5 (*n* = 3; mean 0.04; 95% CI −0.12 to 0.21) compared to TDN scores 1–2 (*n* = 24; mean 0.02; 95% CI −0.07 to 0.12). Changes in EQ-5D 3L index value in subgroups of patients based on TDN scores are shown in **Table 5** and **Figure 4**.

**Table 5:** Change in the EQ-5D 3L index value in subgroups of patients based on TDN scores (*N* = 45).

Cohort (*n* = 45)	Change in EQ-5D 3L index value
Total sample; mean (95% CI)	−0.01 (−0.09 to 0.07)
TDN^1^; mean (95% CI)	
No complications related to surgery (Score 0), *n* = 18	−0.06 (−0.21 to 0.08)
TDN scores 1 to 5; mean (95% CI), *n* = 27	0.03 (−0.06 to 0.11)
* “Mild” complications (TDN 1 and 2), n = 24*	*0.02 (−0.07 to 0.12)*
* “More than mild” complication (TDN 3 to 5), n = 3*	*0.04 (−0.12 to 0.21)*

^1^Therapy-Disability-Neurology.

**Figure 5:**
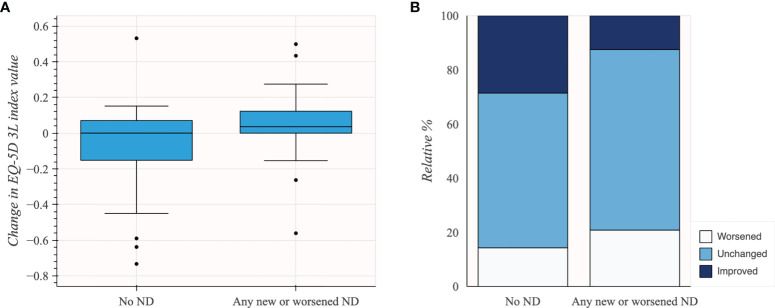
Change in EQ-5D 3L index value in patients grouped by the presence of neurological deficit (ND) at follow-up (*N* = 45). **(A)** Box plot illustrating distribution of change in EQ-5D 3L index values. **(B)** Stacked bar plot illustrating change using MCID groups.

In patients grouped by the presence of neurological deficit at follow-up, patients without new or worsened neurological deficit at follow-up had better HRQoL change with mean −0.08 change in pre- to post-operative EQ-5D 3L index value (*n* = 21; 95% CI −0.20 to 0.05) compared to patients experiencing any new or worsened neurological deficit (*n* = 24; mean 0.05; 95% CI −0.04 to 0.13). Changes in EQ-5D 3L index value in patients grouped by presence of neurological deficit at follow-up are shown in **Table 6** and **Figure 5**.

**Table 6:** Change in the EQ-5D 3L index value in patients grouped by presence of neurological deficit at follow-up (*N* = 45).

Cohort (*n* = 45)	Change in EQ-5D 3L index value
Patients by presence of neurological deficit; mean (95% CI)	
* Any new or worsened neurological deficit^1^, n = 24*	0.05 (−0.04 to 0.13)
* None new or worsened neurological deficit at follow-up, n = 21*	−0.08 (−0.20 to 0.05)

^1^ Including transient or permanent neurological deficits from surgery to the 3-month follow-up.

**Figure 6:**
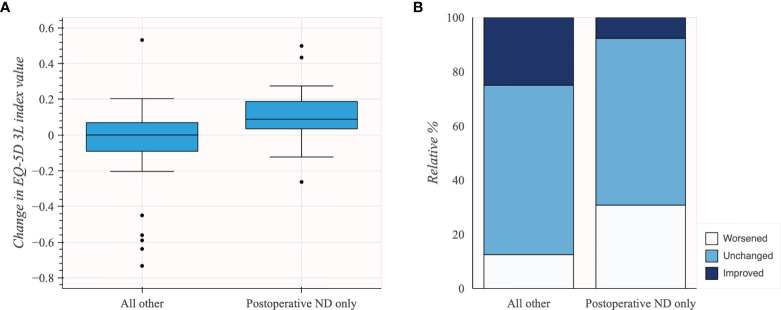
Change in the EQ-5D 3L index value in patients grouped by change in neurological deficit (ND) from admission to follow-up (*N* = 45). **(A)** Box plot illustrating distribution of change in EQ-5D 3L index values. **(B)** Stacked bar plot illustrating change using MCID groups.

Patients with new-onset neurological deficit(s) at follow-up had worse HRQoL change, with mean 0.11 change in pre- to postoperative EQ-5D 3L index value (*n* = 13; 95% CI 0.0 to 0.22) compared to other patients (*n* = 32; mean −0.06; 95% CI −0.15 to 0.03). Changes in EQ-5D 3L index value in patients grouped by change in neurological deficit from admission to follow-up are shown in **Table 7** and **Figure 6**.

**Table 7:** Change in the EQ-5D 3L index value in patients grouped by change in neurological deficit from admission to follow-up (*N* = 45).

Cohort (*n* = 45)	Change in EQ-5D 3L index value
Patients by change neurological deficit^1^; mean (95% CI)	
* Patients with new postoperative neurological deficit only, n = 13*	0.11 (0.00 to 0.22)
All other patients, *n* = 32	−0.06 (−0.15 to 0.03)
* Patients with new post- and with preoperative neurological deficit, n = 11*	*−0.03 (−0.15 to 0.09)*
* Patients without new post- and without preoperative neurological deficit, n = 13*	*−0.05 (−0.20 to 0.10)*
* Patients without new post- and with preoperative neurological deficit, n = 8*	*−0.12 (−0.37 to 0.13)*

^1^New neurological deficits were defined as new or worsened (transient/permanent) from surgery to the 3-month follow-up.

Patients undergoing any adjuvant treatment within 3 months of surgery showed a similar change in EQ-5D 3L index values mean −0.06 (*n* = 28, CI −0.17 to 0.06). Despite small subgroups, no major trend was seen with the different adjuvant treatment (*n* = 11, mean −0.05, CI −0.23 to 0.14 for patients undergoing chemotherapy only; *n* = 8, mean −0.19, CI −0.45 to 0.07 for patients undergoing radiotherapy only; and *n* = 9, mean 0.05, CI −0.09 to 0.19 for patients undergoing chemotherapy and radiotherapy). The patients without any adjuvant treatment within 3 months of surgery had comparable results (*n* = 17, mean 0.07, CI 0.00 to 0.13).

## Discussion

Comparing the TDN score to LIC in our population-based cohort of dLGG, we observed a slight shift from mild complications towards complications of higher order. This shift represented the *“*severity” of the complication that was expressed in either new neurological deficits or new functional deficits. This finding demonstrates the capacity of the TDN score to multidimensionally report the functional consequences and severity of postoperative complications related to dLGG surgery. Regarding HRQoL, no important clinical differences, measured as EQ-5D 3L index values, were found between patients with and patients without complications related to surgery. Although acknowledging that subgroups were small, we conclude that there was no apparent difference in HRQoL change among subgroups of patients with complications of different order.

When compared side by side, only a small fraction of patients experiencing mild complications according to LIC received TDN scores higher than 2 due to impairment in mRS scores. Although the majority of patients with dLGG undergoing surgery did not have a marked reduction in functional capacity as measured by mRS, and consequently little effect on results at the group level, it demonstrates that TDN is capable of identifying such patients. Furthermore, most complications initially classified as Grade 1a in LIC were classified as TDN score 2 due to the presence of various neurological deficits postoperatively, showing that the TDN scoring system is clearly capturing the neurological consequences of surgery. Nevertheless, the diversity of neurological deficits at follow-up is not further differentiated by the TDN scoring system. Overall, when compared to LIC, the trajectories marked by TDN classification, although useful, do not introduce substantial changes into the classification of complications related to surgery in our cohort of patients with dLGG.

Acknowledging limitations of the HRQoL subset size, our data suggest that changes in HRQoL reflect changes in neurological function related to aspects of patient’s daily activities that are not addressed by the TDN score. Given the small sample size, we only used the EQ-5D 3L index value. An analysis of all EQ-5D 3L dimensions or use of a more fine-tuned instrument in a larger cohort may certainly better reflect neurological function than the EQ-5D 3L index value alone. It was previously demonstrated that new neurological deficits can have major undesirable effects on HRQoL (21). Nevertheless, we found that patients with new or worsened neurological deficits and patients with postoperative neurological deficit only were mostly classified as TDN 2. We also found that these patients had generally a decline in HRQoL. We suggest that TDN may be too insensitive to changes in neurological function related to aspects of patient’s daily activities that are important to patients, and perhaps more important than many of the non-neurological complications. Despite small numbers, one nuance in our preliminary data suggests that the presence of postoperative neurological deficit alone might not be as relevant from a patient’s perspective as an unexpected decline in neurological status from admission to follow-up.

Considering the relative short period of time from radiological diagnosis to 3 months follow-up, it would be interesting to explore how this trend in HRQoL evolves in the medium and long term. This would be especially important in patients with oligodendroglioma or *IDH*-mutated astrocytoma where a more indolent course of disease is expected. However, in that case, attention should be also given to the so-called response shift phenomena. It has been reported that a response shift seems to reduce the effects of HRQoL changes in patients with glioma (22). Thus, this is a potential source of unexpected findings and a potential limitation for anchoring outcomes with HRQoL in the longer term. Despite unclear patterns in the short term, the effect of adjuvant treatment on HRQoL change should also be considered in future research.

Some limitations raised by the authors of the TDN classification system are its inability to differentiate between adverse events and failure to cure, and the lack of account for surgical complexity (4). In our study, including some patients with an unfavorable prognosis, we strictly limited the recording of adverse events and follow-up to 1 and 3 months, respectively, to reduce the risk of upgrading complications due to the natural course of the disease or due to the documented side effects (e.g., thrombocytopenia and leukopenia) related to adjuvant treatment. Thus, we can appreciate the importance of time-point measurement tailored towards particular diagnostic groups. A 3-month time interval was shown to be sufficient for recovery from transient deficits following surgery in patients with dLGG (23). However, in patients with a notable deterioration in mRS at follow-up due to tumor progression, mRS measurements could not be used for TDN scoring. Furthermore, in order to avoid too much “thresholding” of own results (with a significant portion of data based on retrospective data), any new or worsened postoperative neurological deficit was carefully recorded. Being cautious not to select deficits considered more important by the clinical team than the patient, or remove expected ones, we were interested in keeping the patient perspective (24). Thus, our study presents a comprehensive, although retrospective, view on the clinical burden related to surgery that patients with dLGG experience.

In neurosurgery, multidimensional PROMs measuring different aspects of HRQoL have shown slight to moderate agreement with traditional clinical scales including KPS and mRS (21, 25, 26). Patient- and surgeon-reported outcomes detect different aspects of the patients’ health status that are relevant for clinical practice, with the potential to enhance individually tailored patient care (27). We believe that the weight of neurological deficit and function within the TDN score should be further explored to reflect the burden of adverse event as experienced by patients, if the intention of TDN indeed is a more holistic adverse event classification. In case the TDN fails to capture the importance of new or worsened neurological deficit(s) and impaired functional status, we would advocate continuing to report these outcomes separately and not hidden within the TDN grade. Further research on HRQoL trajectories in relation to specific complications is needed.

## Strengths and Limitations

The demographics of the study cohort together with the variables associated with presence of complications following glioma surgery were comparable with previous reports on patients with dLGG (28–32), indicating that our results hold high external validity. There are, however, limitations inherent to the retrospective design of our study. Although the inclusion of patients and a portion of the data was prospectively collected, mRS was not included in the collection template. Thus, mRS was supplemented in retrospect. At our institution, clinical routine comprising the period 2017–2020 included the screening of patients by neuropsychological testing. Therefore, a bias towards detecting more cognitive deficits in the prospectively recruited cohort may be present both pre- and postoperatively.

The inclusion of HRQoL enabled us to explore the impact of negative outcomes as reported by patients. However, the limited sample size for HRQoL data did not allow us to perform statistical analyses on the relation between TDN score and EQ-5D 3L. Thus, data exploration in smaller but clinically relevant subgroups was not possible. The EQ-5D 3L index value is known for being prone in particular to the ceiling effect (20). Indeed, there was a significant ceiling effect in our cohort, where the best possible EQ-5D 3L index value at admission was scored by 18% of our patients. In contrast, there was no floor effect for the EQ-5D 3L index value.

## Conclusions

TDN score compared to LIC tends to modestly capture more adverse events of higher order, by putting new emphasis on the functional and neurological outcome. Classification with TDN seems intuitive and adequate to be used in future studies. We suggest that future work on TDN score, with further validation against PROM, should explore if the neurological and functional consequences should be weighed differently.

## Data Availability Statement

The raw data supporting the conclusions of this article will be made available by the authors upon reasonable request, without undue reservation.

## Ethics Statement

The studies involving human participants were reviewed and approved by the Regional Ethical Review Board in Gothenburg, Sweden (Dnr. 1067-16). Informed written consent was obtained for all prospectively included patients. The committee waived the need of written consent for the retrospective cohort. The study was conducted in accordance with the Declaration of Helsinki.

## Author Contributions

All authors participated equally in the initial design and final reviewing of the manuscript. TV contributed to the planning of the study, data curation, data analysis, and drafting of the manuscript, including design of figures and tables, and revising and submission of manuscript. AC contributed by recruiting patients, reviewing the manuscript, and approval of the final manuscript. DB contributed by mRS assessment, reviewing the manuscript, and approval of the final manuscript. IR contributed by HRQoL measurement, reviewing the manuscript, and approval of the final manuscript. AS contributed by reviewing the manuscript and approval of the final manuscript. AJ contributed by planning the study, recruiting patients, reviewing the manuscript, and approval of the final manuscript. All authors contributed to the article and approved the submitted version.

## Funding

AJ holds research grants from the Swedish Research Council (2017-00944) and the agreement between the Swedish government and the county councils (the ALF agreement, ALFGBG-716671). AC has received grants from the Gothenburg Society of Medicine.

## Conflict of Interest

The authors declare that the research was conducted in the absence of any commercial or financial relationships that could be construed as a potential conflict of interest.

## Publisher’s Note

All claims expressed in this article are solely those of the authors and do not necessarily represent those of their affiliated organizations, or those of the publisher, the editors and the reviewers. Any product that may be evaluated in this article, or claim that may be made by its manufacturer, is not guaranteed or endorsed by the publisher.
