# Supplementary material for: Classification of Adverse Events Following Surgery in Patients With Diffuse Lower-Grade Gliomas
Source: Front Oncol. 2021 Dec 21;11:792878. doi: 10.3389/fonc.2021.792878 (PMC8724913; doi:10.3389/fonc.2021.792878)
Supplement: Supplementary file 1 [file Table_1.docx]

**Supplementary Table 1.** Associations between clinical variables at admission with a binary dependent variable for patients with complications related to surgery (N=231).

| **Variable** |  | **Simple logistic regression.**  **Response: Complications related to surgery** | | | |  | **Multiple logistic regression.**  **Response: Complications related to surgery** | | | |
| --- | --- | --- | --- | --- | --- | --- | --- | --- | --- | --- |
|  |  | **Exp(B)** | **95% Wald CI for Exp(B)** | | **P-value^1^** |  | **Exp(B)** | **95% Wald CI for Exp(B)** | | **P-value^1^** |
|  |  |  | **Lower** | **Upper** | **(unadjusted)** |  |  | **Lower** | **Upper** | **(adjusted)** |
| Age at surgery |  | 0.999 | 0.397 | 2.400 | 0.87 |  | 1.007 | 0.984 | 1.3031 | 0.55 |
| Male |  | 1.089 | 0.645 | 1.837 | 0.75 |  | 1.071 | 0.590 | 1.945 | 0.82 |
| KPS^1^ (>=90) |  | 0.997 | 0.594 | 1.673 | 0.99 |  | 0.933 | 0.467 | 1.863 | 0.84 |
| History of epilepsy |  | 1.196 | 0.687 | 2.083 | 0.53 |  | 1.328 | 0.636 | 2.772 | 0.45 |
| Motor deficit |  | 0.478 | 0.207 | 1.108 | 0.09 |  | 0.422 | 0.140 | 1.275 | 0.13 |
| Cognitive deficit |  | 2.571 | 1.246 | 5.305 | 0.01 |  | 3.701 | 1.415 | 9.676 | 0.01 |
| Visual deficit |  | 0.533 | 0.156 | 1.822 | 0.32 |  | 0.486 | 0.104 | 2.278 | 0.36 |
| Language deficit |  | 1.828 | 0.816 | 4.097 | 0.14 |  | 2.175 | 0.735 | 6.434 | 0.16 |
| Tumor resection as choice of primary surgery intervention |  | 3.812 | 1.828 | 7.948 | <0.001 |  | 6.181 | 2.325 | 16.435 | <0.001 |
| WHO 2016 classification: |  |  |  |  |  |  |  |  |  |  |
| Oligodendroglioma, WHO grade 2 |  | 1.120 | 0.550 | 2.280 | 0.76 |  | - | - | - | Reference |
| Oligodendroglioma, WHO grade 3 |  | 1.041 | 0.498 | 2.177 | 0.91 |  | 0.803 | 0.281 | 2.291 | 0.68 |
| Diffuse astrocytoma, IDH-mutant, WHO grade 2 |  | 1.277 | 0.627 | 2.604 | 0.50 |  | 1.138 | 0.397 | 3.263 | 0.81 |
| Astrocytoma, IDH-mutant, WHO grade 3 |  | 1.193 | 0.599 | 2.376 | 0.62 |  | 0.808 | 0.281 | 2.324 | 0.69 |
| Diffuse astrocytic glioma, IDH-wildtype, WHO grade 2 |  | 0.554 | 0.286 | 1.074 | 0.08 |  | 0.852 | 0.282 | 2.570 | 0.78 |
| Diffuse astrocytic glioma, IDH-wildtype, WHO grade 3 |  | 1.122 | 0.568 | 2.219 | 0.74 |  | 0.883 | 0.289 | 2.689 | 0.83 |
| Main tumor location: |  |  |  |  |  |  |  |  |  |  |
| Frontal |  | 1.097 | 0.654 | 1.839 | 0.73 |  | - | - | - | Reference |
| Temporal |  | 1.050 | 0.593 | 1.859 | 0.87 |  | 0.943 | 0.431 | 2.066 | 0.88 |
| Parietal |  | 1.000 | 0.407 | 2.455 | 1.00 |  | 0.968 | 0.324 | 2.887 | 0.95 |
| Occipital |  | 1.101 | 0.068 | 17.815 | 0.95 |  | 0.700 | 0.028 | 17.525 | 0.83 |
| Insular |  | 0.680 | 0.254 | 1.820 | 0.44 |  | 0.645 | 0.199 | 2.150 | 0.49 |
| Basal ganglia |  | 0.728 | 0.119 | 4.443 | 0.73 |  | 1.788 | 0.148 | 21.640 | 0.65 |
| Tumor located in eloquent regions (UCSF^4^) |  | 1.339 | 0.764 | 2.341 | 0.31 |  | 2.885 | 1.410 | 5.902 | 0.01 |
| Tumor volume^5^ |  | 0.998 | 0.995 | 1.001 | 0.22 |  | 0.996 | 0.991 | 1.001 | 0.08 |

^1^ Statistical significance level was set to p < .05. Collinearity was not found between the variables included in the analysis.

^1^ Karnofsky Performance Status Scale. Recategorized into <90 and >=90 for the purposes of this analysis.

^4^ University of California San Francisco classification system.

^5^ Volume in cubic millimeters. One missing case due to unavailable MRI.
